# The influence of residual force enhancement on spinal and supraspinal excitability

**DOI:** 10.7717/peerj.5421

**Published:** 2018-08-03

**Authors:** Caleb T. Sypkes, Benjamin J. Kozlowski, Jordan Grant, Leah R. Bent, Chris J. McNeil, Geoffrey A. Power

**Affiliations:** 1 Department of Human Health and Nutritional Sciences, College of Biological Sciences, University of Guelph, Guelph, ON, Canada; 2 School of Health and Exercise Sciences, University of British Columbia, Kelowna, BC, Canada

**Keywords:** Integrated electromyography, Residual force enhancement, Transcranial magnetic stimulation, Cervicomedullary electrical stimulation, History-dependence of force, Eccentric, Performance enhancing, Dorsiflexion, Tibialis anterior

## Abstract

**Background:**

Following active muscle lengthening, there is an increase in steady-state isometric force as compared with a purely isometric contraction at the same muscle length and level of activation. This fundamental property of skeletal muscle is known as residual force enhancement (RFE). While the basic mechanisms contributing to this increase in steady-state isometric force have been well documented, changes in central nervous system (CNS) excitability for submaximal contractions during RFE are unclear. The purpose of this study was to investigate spinal and supraspinal excitability in the RFE isometric steady-state following active lengthening of the ankle dorsiflexor muscles.

**Methods:**

A total of 11 male participants (20–28 years) performed dorsiflexions at a constant level of electromyographic activity (40% of maximum). Half of the contractions were purely isometric (8 s at an ankle angle of 130°), and the other half were during the RFE isometric steady-state following active lengthening (2 s isometric at 90°, a 1 s lengthening phase at 40°/s, and 5 s at 130°). Motor evoked potentials (MEPs), cervicomedullary motor evoked potentials (CMEPs), and compound muscle action potentials (M-waves) were recorded from the tibialis anterior during the purely isometric contraction and RFE isometric steady-state.

**Results:**

Compared to the purely isometric condition, following active lengthening, there was 10% RFE (*p* < 0.05), with a 17% decrease in normalized CMEP amplitude (CMEP/M_max_) (*p* < 0.05) and no change in normalized MEP amplitude (MEP/CMEP) (*p* > 0.05).

**Discussion:**

These results indicate that spinal excitability is reduced during submaximal voluntary contractions in the RFE state with no change in supraspinal excitability. These findings may have further implications to everyday life offering insight into how the CNS optimizes control of skeletal muscle following submaximal active muscle lengthening.

## Introduction

Residual force enhancement (RFE) is the increase in isometric steady-state force following active muscle lengthening when compared to a purely isometric contraction at the same muscle length and level of activation (Abbott & Aubert, 1952; Power, Rice & Vandervoort, 2012; Seiberl et al., 2015). This history-dependent property of muscle has been observed in vitro from the sarcomere to the whole muscle level, and in vivo during both electrically stimulated as well as maximal and submaximal voluntary contractions in humans (Lee & Herzog, 2002; Hahn et al., 2012; Power, Rice & Vandervoort, 2012; Herzog et al., 2016; Joumaa, Fitzowich & Herzog, 2017; Chapman et al., 2018). While there have been many investigations into the basic underlying mechanisms of RFE (Lee, Joumaa & Herzog, 2007; Joumaa et al., 2008; Koppes, Herzog & Corr, 2013; Power et al., 2013; Herzog et al., 2016), the implications of this history-dependent phenomenon on voluntary control of force production remain unknown. More specifically, it is unclear how spinal and supraspinal excitability are modulated in the isometric force enhanced steady-state during submaximal voluntary contractions.

Lengthening (eccentric) contractions typically produce more force than isometric contractions, and are associated with lower muscle activation as indicated by root mean square electromyography (EMG_RMS_) amplitude (Duchateau & Enoka, 2016). The lower EMG_RMS_ observed during lengthening contractions has been attributed to a reduction in motor unit recruitment and firing rate compared with isometric contractions (Howell et al., 1995; Altenburg et al., 2009). This reduction in motor unit recruitment and firing rate may indicate a lower level of activation distributed across the entire motor neuron population, or the activation of only a subset of the entire population during active lengthening contractions (Enoka, 1996). A reduction in muscle activation also applies to the force enhanced isometric steady-state following active lengthening. When matching force output, EMG in the force enhanced isometric steady-state is typically lower than that of a purely isometric contraction under both submaximal and maximal conditions (Lee & Herzog, 2002; Oskouei & Herzog, 2005; Seiberl et al., 2015; Jones, Power & Herzog, 2016), suggesting that spinal or supraspinal factors may be involved in actively limiting central drive under conditions of enhanced force production capacity.

Modulation of central nervous system (CNS) excitability occurs during (Gruber et al., 2009) and following (Hahn et al., 2012) lengthening contractions. Gruber et al. (2009) found reduced motor evoked potential (MEP) and cervicomedullary motor evoked potential (CMEP) amplitudes, and an increase in the MEP to CMEP ratio for the biceps brachii during maximal eccentric compared to isometric contractions of the elbow flexors. However, during submaximal contractions, only the CMEP amplitude decreased. These findings indicate a reduction in spinal excitability (i.e., CMEP amplitude), and increased supraspinal excitability (i.e., MEP to CMEP ratio) during maximal active muscle lengthening, and a reduction in spinal excitability during submaximal eccentric contractions (Gruber et al., 2009). In the RFE isometric steady-state following maximal intensity lengthening contractions of the plantar flexors, Hahn et al. (2012) showed increased supraspinal excitability and no change in spinal excitability (increased MEP amplitude and unchanged CMEP amplitude) compared to the purely isometric condition. As well, there was a trend toward greater V-wave amplitude, suggesting enhanced motor neuron output or increased stretch reflex excitability during RFE (Hahn et al., 2012). Submaximal contractions more closely represent everyday movements and could offer further insight into voluntary control of force in the RFE steady-state. It is currently unknown whether alterations in CNS excitability observed in previous studies are present following submaximal intensity lengthening contractions in the RFE state.

The purpose of the present study was to investigate spinal and supraspinal excitability in the isometric steady-state following submaximal intensity lengthening contractions. Similar to submaximal intensity lengthening conditions, it was hypothesized that in the RFE isometric steady-state, increased torque during a submaximal contraction would be accompanied by increased supraspinal excitability and reduced spinal excitability, as indicated by increased normalized MEP amplitude and reduced normalized CMEP amplitude.

## Methods

### Participants

A total of 11 healthy male participants with a mean ± standard deviation (SD) age of 24 ± 2 years, height of 177 ± 4 cm, and mass of 75 ± 7 kg were recruited from the university population for participation in the study. All had no prior history of neuromuscular disease or ankle joint injuries. Data were collected within a single testing session. Participants gave written informed consent prior to testing and all procedures were approved by the Human Research Ethics Board of the University of Guelph (REB: 15NV008).

### Experimental set-up

The following methods are used frequently in our lab and there is overlap in the language used throughout this methods section to that of Sypkes et al. (2017). A HUMAC NORM dynamometer (CSMi Medical Solutions, Stoughton, MA, USA) was used for all torque, angular velocity, and position recordings. Each participant sat with their right hip and knee angles set at 110° and 140° (180°; straight), respectfully. Joint angles were measured using a goniometer. The right knee was immobilized with the dynamometer’s leg restraint (superior) and a malleable cushion (inferior), while movement at the torso was restricted with a four-point seatbelt harness. The right foot was fixed to a dorsi/plantar flexor adaptor with one inelastic strap placed over the ankle and another at the mid-distal portion of the metatarsals. The maximal ankle dorsiflexion and plantar flexion angles were set to 90° and 130° (90°; neutral), respectively, allowing for 40° of ankle excursion.

Locations for the EMG electrodes were prepared by shaving and cleaning the skin with alcohol swabs. Silver–silver chloride (Ag-AgCl) electrodes (1.5 × 1 cm: Kendall, Mansfield, MA, USA) were used for all recordings. The active electrode was placed over the tibialis anterior approximately seven cm inferior and two cm lateral to the tibial tuberosity, and a reference electrode was placed over the distal tendon of the tibialis anterior, at the level of the malleoli. To record antagonist muscle activation, the active electrode was placed on the soleus, along the midline of the leg approximately two cm inferior to the border of the heads of the gastrocnemii, and a reference electrode was placed on the calcaneal tendon. A single ground electrode was centered on the patella.

**Figure 1:**
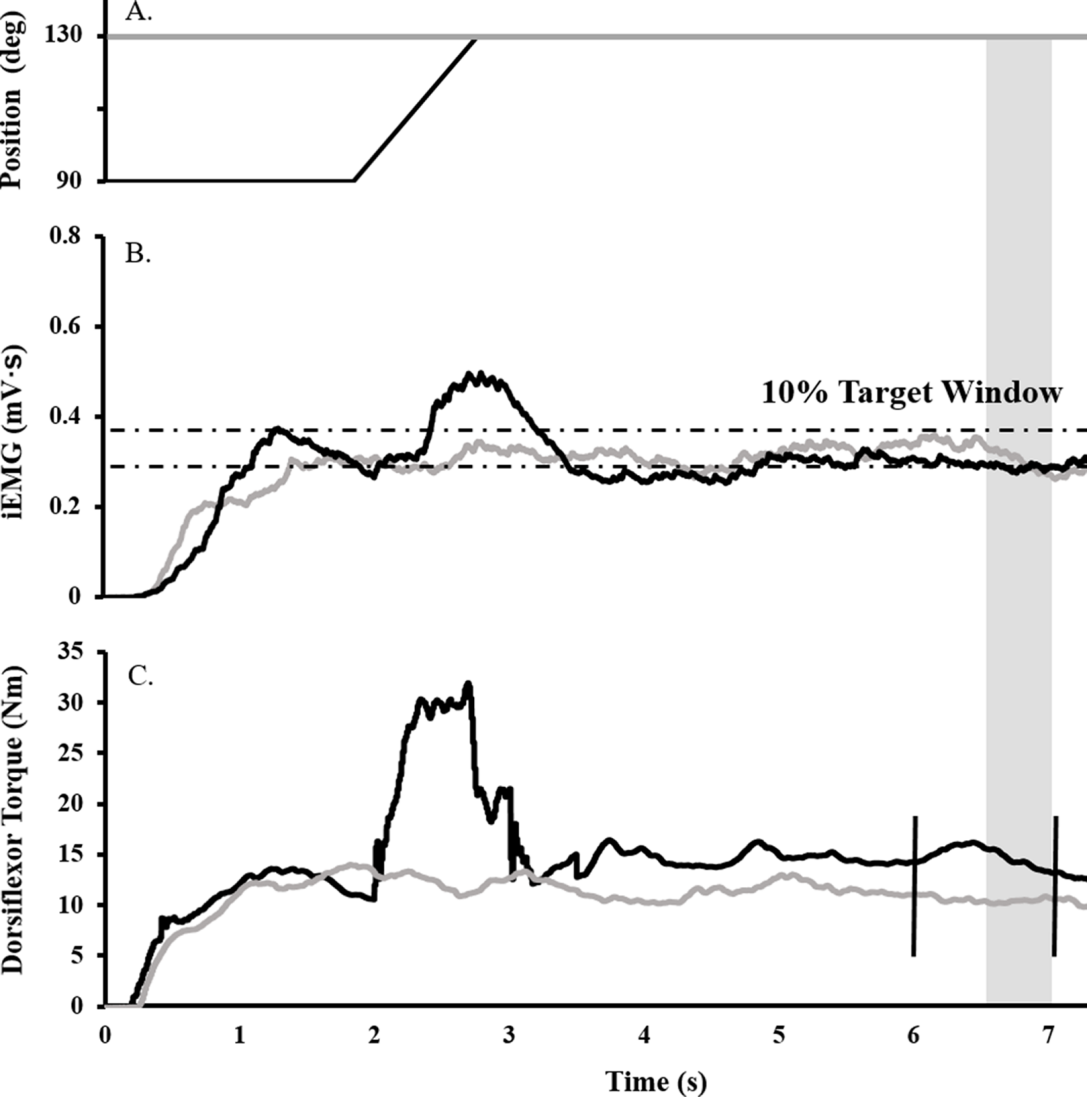
Raw data trace showing experimental procedure. Ankle angle (A), tibialis anterior iEMG (B), and dorsiflexor torque (C) traces during RFE (black) and ISO (gray) contractions for a representative participant. During RFE trials, a contraction corresponding to 40% iEMG was initiated for 2 s at 90° PF before the dynamometer arm rotated the ankle at 40°/s to an ankle angle of 130° PF. A maximal stimulus was delivered to the deep fibular nerve (vertical black line ) at the sixth second of the contraction (to elicit an M_max_), while a TMS or CMS pulse was administered at the seventh second (to elicit an MEP or CMEP, respectively). RFE was determined over a 500 ms window (shaded gray). During isometric reference trials, the same protocol was in effect, with the exception that the ankle angle was fixed at an angle at 130° PF.

Surface EMG, torque, angular velocity, joint angle, and stimulus trigger data were converted to digital format using a 12-bit analog-to-digital converter (PowerLab System 16/35; ADInstruments, Bella Vista, NSW, Australia), and analyzed with Labchart software (Labchart, Pro Modules 2014, v. 8). Torque and EMG data were recorded at a sampling rate of 1,000 and 2,000, respectively. EMG data were band pass filtered using a digital filter (3–1,000 Hz). Figure 1 depicts the joint angle, integrated EMG (iEMG), and torque traces for a single trial.

#### Deep fibular and tibial nerve stimulation

**Figure 2:**
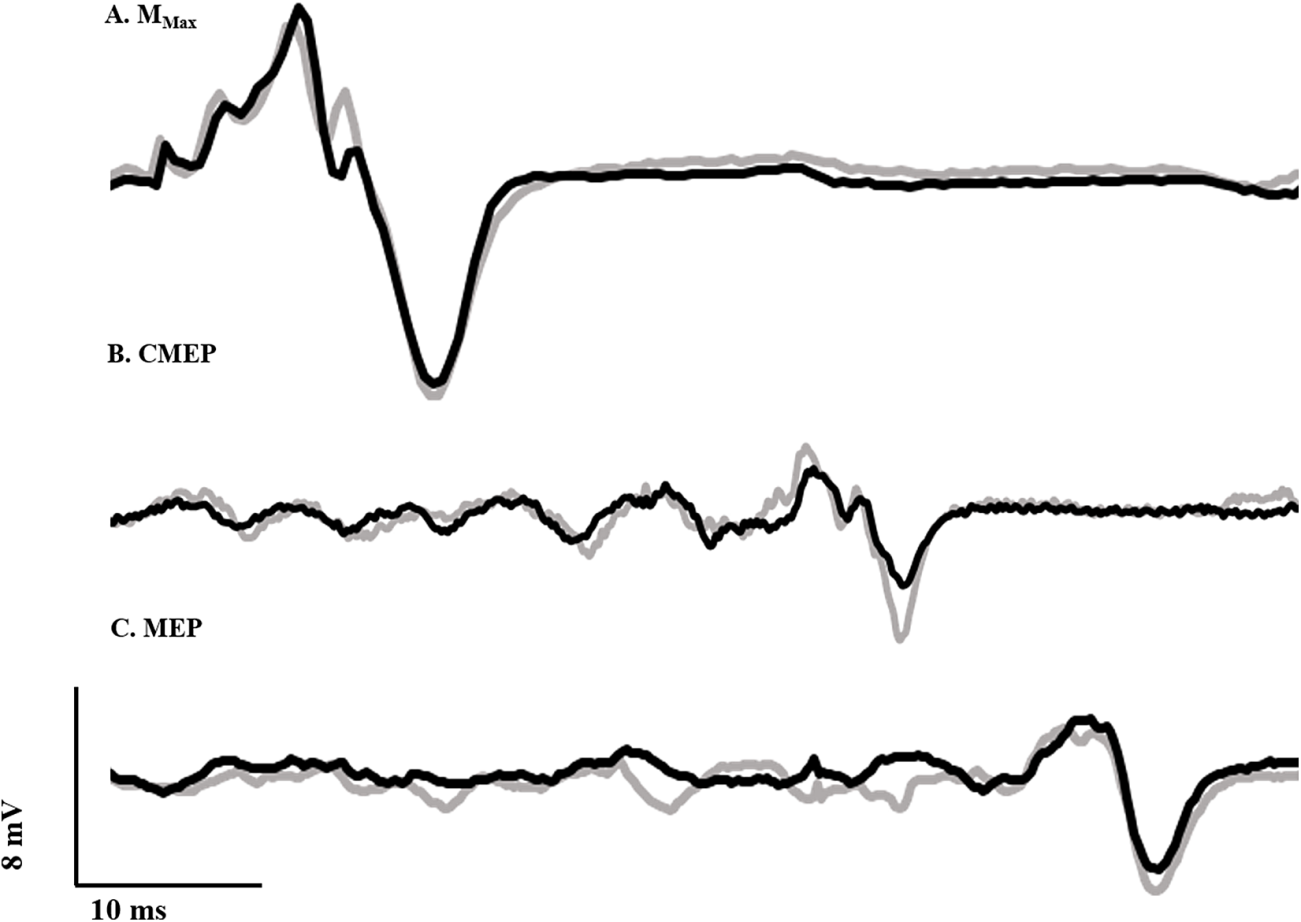
Evoked potential raw traces. Single raw data traces of an M_max_ (A), CMEP (B), and MEP (C) recorded from the tibialis anterior following deep fibular nerve stimulation, CMS and TMS, respectively, in the RFE (black) and ISO (gray) states.

To normalize voluntary EMG and evoked potentials, maximal compound muscle action potentials (M-waves) were recorded over the tibialis anterior (Fig. 2A) and soleus muscles by transcutaneously stimulating the deep fibular and tibial nerves, respectively, with a standard clinical bar electrode (Empi, St. Paul, MN, USA) coated in conductive gel. The deep fibular nerve was located by palpating the head of the fibula and moving posteroinferiorly until the nerve was intercepted. The tibial nerve, innervating the plantar flexor muscles, was found by locating the distal tendon of the semitendinosus muscle and moving laterally while palpating deep into the popliteal fossa. All peripheral nerve stimuli were delivered as a single pulse from a constant current, high voltage stimulator (model DS7AH; Digitimer, Welwyn Garden City, Hertfordshire, UK). Voltage was set to a maximum of 400 V and pulse width to 200 μs. Current was increased until a plateau was reached for the peak-to-peak amplitude of the M-wave (M_max_). To ensure consistent activation of all motor units throughout the experiment, the current (60–150 mA) was adjusted to a supramaximal level, equivalent to 110% of that required to generate M_max_.

#### Maximum voluntary contraction and voluntary activation

Voluntary activation of the dorsiflexors was assessed during brief maximum voluntary contractions (MVCs) performed both prior to and following the experimental protocol. The interpolated twitch technique was used to evaluate voluntary activation during MVCs (Belanger & McComas, 1981; Cheng et al., 2013). The torque resulting from a stimulus to the deep fibular nerve presented during the plateau phase of the MVC was compared to a resting twitch evoked 1–2 s after relaxation. The level of voluntary activation was calculated as: voluntary activation (%) = [1–(interpolated twitch torque/resting twitch torque)] × 100%. Participants were verbally encouraged during all MVCs and the torque trace was visible throughout all contractions (Gandevia, 2001). All participants were required to reach a minimum of 95% voluntary activation, and were given 5 min of rest before continuing with the experiment. All participants were capable of achieving ≥95% with one to two attempts.

#### Determining submaximal muscle activation

To determine the submaximal iEMG target for the activation matching contractions, as described previously (Sypkes et al., 2017), participants were instructed to perform an 8 s maximal dorsiflexion contraction at an ankle angle of 90°. The average iEMG collected between 5.5 and 6.5 s was then used to determine the 40% submaximal iEMG target. A ± 5% window was calculated about this 40% target, and for all subsequent activation-controlled contractions, participants were instructed to maintain their iEMG within guidelines marking this target window (Fig. 1B).

#### Cervicomedullary stimulation

Ag/AgCl electrodes (10 mm diameter—Cleartrace 1700-030; ConMed Corporation, Utica, NY, USA) were used for cervicomedullary stimulation (CMS) to generate CMEPs by passing a current across the spinal cord at the level of the mastoids. Electrodes were placed at a location approximately two cm superior and medial to the mastoid process (Ugawa et al., 1991). Single pulse stimuli were presented (anode on right side and cathode on left) with a constant current, high voltage stimulator (model DS7AH; Digitimer, Welwyn Garden City, Hertfordshire, UK). Voltage was set to a maximum of 400 V and pulse width to 200 μs. Current was adjusted in order to produce a CMEP from the tibialis anterior with a peak-to-peak amplitude equivalent to approximately 40% of resting M_max_ (Fig. 2B), while the participant performed a brief isometric contraction at 40% iEMG at an ankle angle of 90°. This current (150–325 mA) was used for the remainder of the experiment. A CMEP of 40% M_max_ was selected to match our recent work (Grant et al., 2017; Sypkes et al., 2017) and probe the excitability of low and high threshold motoneurons.

#### Transcranial magnetic stimulation

Motor evoked potentials were elicited by transcranial magnetic stimulation (TMS) of the motor cortex. Stimulation was delivered with a double cone coil (110 mm) linked to two Magstim 200^2^ stimulators via a BiStim module (Magstim, Dyfed, UK). In order to determine the ideal location for coil placement, single stimuli were delivered at 20% of stimulator output while the participant performed brief control contractions at 40% iEMG. The coil was originally placed at the vertex, and was moved in one cm increments to the left as well as forward and backward. Limb dominance was not assessed, and the right ankle dorsiflexors were tested in all subjects. The placement which yielded the largest MEP was marked on the participant’s scalp and used throughout the duration of testing. Stimulus intensity was adjusted until the MEP amplitude was equivalent to that of the CMEP (i.e., ∼40% of resting M_max_; Fig. 2C) during a brief contraction at 90° ankle angle corresponding to 40% iEMG. This stimulus intensity (25–65% of stimulator output) was used for the remainder of the experiment.

### Experimental procedures

Each isometric control trial (ISO) was followed by an RFE trial. Protocol A was followed by protocol B (details below) and this sequence was repeated a total of four times. Thus, four ISO trials and four RFE trials were performed for each of the two protocols for a total of 16 contractions matching an iEMG target of 40% (Fig. 1B). Participants were given visual feedback of the iEMG trace on the computer monitor and were verbally encouraged to match the target as closely as possible during all contractions. Three minutes of rest separated all submaximal contractions throughout the experiment.

#### Protocol “A”: eliciting the MEP and M_max_

For each RFE trial, the protocol consisted of a 40% iEMG contraction involving a 2 s isometric phase with the ankle at 90°, a 1 s isokinetic lengthening phase (40°/s) and a 5 s isometric phase at 130°. An electrical stimulus was delivered to the deep fibular nerve at the sixth second (time point 1) and the TMS pulse was administered at the seventh second (time point 2) of the 8 s contraction. For the ISO trials, the ankle was set to an angle of 130°. An isometric submaximal contraction at 40% iEMG was performed for 8 s with stimulation of the deep fibular nerve occurring at the sixth second (time point 1) and the TMS pulse at the seventh second (time point 2) of the trial.

#### Protocol “B”: eliciting the CMEP

The contraction parameters were identical to those of Protocol A, but the type of stimulation differed. CMS was administered at the seventh second (time point 2) of each trial to correspond with the administration of TMS in protocol A.

### Data analysis and statistics

The mean torque and root mean squared EMG (EMG_RMS_) were calculated in a 500 ms window occurring prior to each stimulus. A paired *t-*test was performed to compare the torque and EMG data between RFE and ISO trials for each stimulus to validate the presence of RFE at the time of stimulation. With respect to RFE, non-responders were identified as participants who displayed no increase in isometric steady-state torque following active lengthening as compared with the purely isometric contraction (i.e., RFE). When no RFE occurred during MEP and CMEP trials, all corresponding steady-state isometric and purely isometric torque, raw and normalized tibialis anterior and soleus EMG, M_max_, CMEP, and MEP potential data were removed from data analysis (*n* = 2 subjects). While non-responders are common in most investigations of RFE, it remains unclear why some individuals do not display RFE (Seiberl et al., 2015). It has, however, been hypothesized that differences in muscle fiber types, the magnitude of muscle length change during stretch, and titin isoforms may contribute to differences between responders and non-responders (Joumaa et al., 2008; Seiberl et al., 2015). Additionally, one subject was removed for experimenters failing to match MEP peak to peak amplitude to 40% of their M_max_. To assess spinal excitability in the RFE and ISO states, CMEPs were normalized to M_max_ (CMEP/M_max_) to control for possible changes in peripheral excitability. To measure supraspinal excitability in the RFE and ISO states, MEPs were normalized to CMEPs (MEP/CMEP) to control for any changes in both spinal and peripheral excitability. The EMG_RMS_ of the resting M_max_ recorded at the tibialis anterior and soleus was used to normalize voluntary EMG for each muscle. The EMG_RMS_ of the soleus M_max_ was also used to quantify antagonist coactivation.

In order to detect and subsequently remove outlier data for each participant, a mean value was created for normalized CMEPs and MEPs and any individual response which fell more than two SDs above or below the mean was rejected. A paired *t* test was used to test for differences in M_max_ (mV), CMEP (% M_max_), and MEP (% CMEP) data between the RFE and ISO states to elucidate RFE-induced changes in peripheral, spinal, and supraspinal excitability, respectively. In order to assess any effects of fatigue during the experimental protocol, a paired *t* test was also used to compare torque produced during the MVC performed before and after the experiment. Effect sizes are reported as Cohen’s *d*. Descriptive data found in text are reported as means ± SD, and presented in figures as means ± standard error of the mean. Significance was determined based on a *p*-value of < 0.05.

## Results

### Maximum voluntary contraction torque and voluntary activation

Pre-trial MVC torque was 24.4 ± 5.2 Nm, and all participants achieved near-maximal values for voluntary activation (99.6% ± 0.6%). Following the experimental protocol, MVC torque and voluntary activation were reassessed. The post-trial MVC torque (22.7 ± 6.0 Nm) was not significantly different from the pre-trial value (paired *t*: d*f* (10), *t*-value (−2.01), *p* = 0.07, *d_z_* = 0.60), and all participants remained capable of achieving near-maximal voluntary activation values (98.7% ± 1.7%).

### Dorsiflexion torque and muscle activity in the RFE state following active lengthening

**Figure 3:**
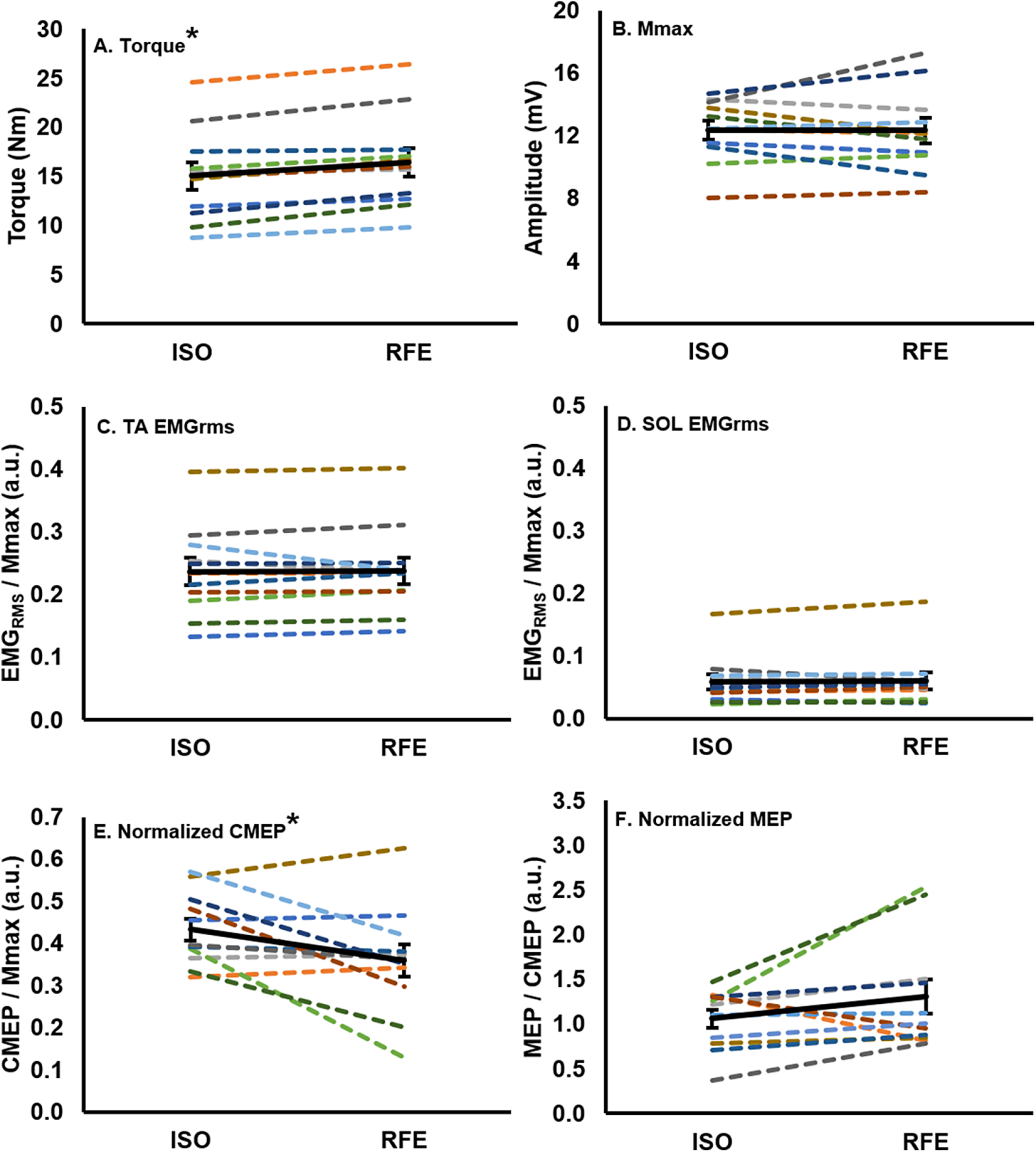
Mean values for each participant and the group mean. Mean values for each participant (colored lines) and the group (*n* = 11) mean (black line; error bars indicate standard error of the mean) in the RFE and ISO states. Each participant maintains the same color across all graphs. There was a ∼10% increase in torque (A), and a ∼17% decrease in normalized CMEP (E) in the RFE state when compared with the ISO state (*p < 0.05). There was no significant difference in EMG_RMS_ recorded from the tibialis anterior (C) or soleus (D), M_max_ (B), or normalized MEP (F) between the two states (*p* > 0.05).

Following active lengthening, steady-state isometric torque was significantly greater (paired *t*: d*f* (10), *t*-value (−5.89), *p* = 0.00015, *d_z_* = 2.02) than the torque recorded during purely isometric contractions at the same muscle length (Fig. 3A), resulting in an average RFE across all participants of 10.0% ± 6.7%. Participants successfully maintained the iEMG target level such that EMG of the tibialis anterior did not differ prior to stimulation during the RFE and ISO contractions (paired *t*: d*f* (10), *t*-value (−0.28), *p* = 0.79, *d_z_* = 0.08), indicating that motor neuron output was similar in both the RFE and ISO states. Antagonist coactivation (i.e., soleus) was not different (paired *t*: d*f* (10), *t*-value (−0.35), *p* = 0.73, *d_z_* = 0.12) between the RFE and ISO contractions, respectively (Figs. 3C and 3D).

### Evoked muscle responses in the residual force enhanced state following active lengthening

There was no significant difference (paired *t*: d*f* (10), *t*-value (0.09), *p* = 0.93, *d_z_* = 0.04) in M_max_ peak-to-peak amplitude between the RFE and ISO state (Fig. 3B). During the ISO state, the peak-to-peak amplitudes of CMEPs and MEPs were successfully matched to 43.3% ± 8.7% and 41.6% ± 11.9% of the resting M_max_, respectively, and were not significantly different from each other (paired *t*: d*f*(10), *t*-value(0.54), *p* = 0.60, *d_z_* = 0.16). When CMEPs were normalized to M_max_ (CMEP/M_max_), there was a significant 17.1% ± 24.8% decrease in peak-to-peak amplitude during the RFE state when compared with the ISO state (paired *t*: d*f* (10), *t*-value (2.32), *p* = 0.04, *d_z_* = 0.70; Fig. 3E). When MEPs were normalized to CMEPs (MEP/CMEP), there was no significant difference (paired *t*: d*f* (10), *t*-value (−1.58), *p* = 0.15, *d_z_* = 0.48) in the mean peak-to-peak amplitudes between RFE and ISO contractions (Fig. 3F).

## Discussion

The current study investigated changes in spinal and supraspinal excitability of the tibialis anterior during the isometric steady-state following active lengthening contractions as compared with a purely isometric contraction at the same muscle length and level of activation (i.e., 40% iEMG activation matching task). The activation matching task was successful in eliciting RFE (10% increase in torque compared to ISO), and the hypothesis was supported by a 17% decrease in spinal excitability (CMEP normalized to M_max_). However, in contrast to our hypothesis, there was no change in supraspinal excitability (MEP normalized to CMEP). Supraspinal and spinal excitability in the force-enhanced isometric steady-state appears to differ when contractions are maximal (Hahn et al., 2012) compared to submaximal (present study). Altered spinal excitability associated with RFE would indicate that, while RFE is indeed an intrinsic fundamental property of skeletal muscle, this history-dependent property of muscle has the potential to influence submaximal force production during voluntary contractions.

### CNS excitability in RFE state

Following active lengthening as compared with a purely isometric contraction, when matching force output, there is a reduction (range: 5–20%) in the activation required to maintain force production as assessed via surface EMG (Oskouei & Herzog, 2005; Seiberl et al., 2012; Jones, Power & Herzog, 2016). From a mechanical perspective, in the force enhanced isomeric steady-state, there is a relatively greater contribution of passive force to total force production, possibly owing to stiffening of the giant molecular spring titin (Herzog, 2018). As a result of this property of muscle, during torque- and activation-matching experiments, the RFE EMG-torque relationship is shifted to the right as compared with a purely isometric contraction (Paquin & Power, 2018), indicating lower activation required to achieve a given torque output. Reduced activation indicates a down-regulation of motor neuron output during the isometric steady-state following active muscle lengthening to achieve the same force level as a purely isometric contraction. This activation reduction in the RFE state has been attributed to a reduction in the number of active motor units (Altenburg et al., 2008) or a reduction in motor unit firing rate (Jakobi et al., 2018). Important to the present study is the use of an activation matching task (i.e., activation clamp). By matching activation, despite the same motor neuron output (40% EMG) for the purely isometric contraction and the isometric steady-state following active lengthening, we were able to show 10% RFE and a 17% reduction in spinal excitability. These findings provide support that the history-dependence of torque alters corticospinal excitability through a neuromechanical coupling (Hahn et al., 2012; Grant et al., 2017; Sypkes et al., 2017). However, there was no change in supraspinal excitability. These findings differ from Hahn et al. (2012) which showed, for the soleus muscle, an increase in supraspinal excitability and no change in spinal excitability following maximal effort lengthening contractions in the RFE state. The divergent findings of the present study and Hahn et al. (2012), may be attributed to the task-dependent nature of corticospinal excitability (For review see Kalmar, 2018). Corticospinal excitability has been shown to differ across contraction intensities (Martin, Gandevia & Taylor, 2006; Oya, Hoffman & Cresswell, 2008; McNeil et al., 2011) and for different muscle groups (Hoffman et al., 2009; Giesebrecht et al., 2010). Thus, it is possible there are task- (maximal vs. submaximal) and muscle-dependent (soleus vs. tibialis anterior) alterations to corticospinal excitability in the RFE state of varying contraction intensities, which requires further investigation.

While the results of the present study diverge from the findings of Hahn et al. (2012), they fall in line with results from other previous studies investigating the influence of altered muscle torque production capacity on corticospinal excitability. When torque production capacity is increased during submaximal lengthening contractions, Gruber et al. (2009) found that when compared to isometric contractions, there is a reduction in spinal excitability (i.e., CMEP amplitude). Lengthening contractions were also associated with increased supraspinal excitability (i.e., MEP/CMEP ratios). In contrast, following active muscle shortening in the torque depressed state, when the ability of the muscle to generate force is impaired, we recently demonstrated increased spinal excitability (i.e., normalized CMEP amplitude) and no change in supraspinal excitability (i.e., normalized MEP amplitude) (Sypkes et al., 2017). The present study, together with the previously mentioned investigations, provides strong evidence for a neuromechanical coupling that may alter corticospinal excitability in a manner that may be related to the capacity of the muscle to produce force.

### Tension-dependent spinal inhibitory mechanisms

We observed a decrease in spinal excitability in the isometric steady-state following active lengthening contractions as compared with the purely isometric contraction. Given the task was activation matched at 40% EMG for both conditions, thus achieving a similar motor neuron output, the alteration to spinal excitability was most likely owing to peripheral sensory inputs. Ib afferents are anatomically located in series with the muscle and aponeurosis, and provide inhibitory sensory feedback to the agonist motor neuron pool during tonic, non-locomotor tasks (Conway, Hultborn & Kiehn, 1987; McCrea et al., 1995). Firing of Ib afferents is modulated in a tension-dependent manner. Therefore, in the present study, the smaller normalized CMEP in the RFE compared to ISO state may be attributed to greater Ib afferent firing due to the increased isometric force after muscle lengthening. Nevertheless, due to the nature of maintaining a constant level of muscle activation, spinal inhibition would be expected to be paired with an increase in supraspinal excitability (i.e., increased normalized MEP amplitude) to counteract inhibition from afferent muscle feedback. While this counterbalance was not observed, there are several subjects in the present study who show a negative relationship between their normalized CMEP and MEP amplitudes, such that an increase or decrease in CMEP amplitude was accompanied by an opposite change in MEP amplitude (Figs. 3E and 3F). Although not statistically significant, this relationship between supraspinal and spinal excitability is similar to what was reported for maximal (Grant et al., 2017) and submaximal torque depression (Sypkes et al., 2017) studies.

## Conclusion

Residual force enhancement, a history-dependent property of muscle, was present during a submaximal activation matching task. In this force enhanced isometric steady-state following an active lengthening contraction, there was a reduction in spinal excitability as compared with a purely isometric contraction. This study adds to a growing body of literature that the history-dependence of force, once thought only to be associated with muscle, has the potential to influence corticospinal excitability. These findings may have further implications for everyday dynamic muscle movements regarding how the CNS optimizes control of skeletal muscle following active muscle lengthening.

## Supplemental Information

10.7717/peerj.5421/supp-1 Supplemental Information 1 Dataset.

## Additional Information and Declarations

### Data Availability

The following information was supplied regarding data availability: The raw measurements are provided in a Supplemental File.
